# *IL-9* and *IL-10* Single-Nucleotide Variants and Serum Levels in Age-Related Macular Degeneration in the Caucasian Population

**DOI:** 10.1155/2021/6622934

**Published:** 2021-04-12

**Authors:** Alvita Vilkeviciute, Dzastina Cebatoriene, Loresa Kriauciuniene, Reda Zemaitiene, Rasa Liutkeviciene

**Affiliations:** ^1^Neuroscience Institute, Lithuanian University of Health Sciences, Medical Academy, Eiveniu St. 2, Kaunas, Lithuania LT-50161; ^2^Department of Ophthalmology, Lithuanian University of Health Sciences, Medical Academy, Eiveniu St. 2, Kaunas, Lithuania LT-50161

## Abstract

Considering the immunological impairment in age-related macular degeneration (AMD), we aimed to determine the associations of *IL-9* rs1859430, rs2069870, rs11741137, rs2069885, and rs2069884 and *IL-10* rs1800871, rs1800872, and rs1800896 polymorphisms and their haplotypes, as well as the serum levels of IL-9 and IL-10 with AMD. 1209 participants were enrolled in our study. SNPs were genotyped using TaqMan SNP genotyping assays by real-time PCR method. IL-9 and IL-10 serum levels were evaluated using ELISA kits. Our study results have shown that haplotypes A-G-C-G-G and G-A-T-A-T of *IL-9* SNPs are associated with the decreased odds of early AMD occurrence (*p* = 0.035 and *p* = 0.015, respectively). A set of rare haplotypes was associated with the decreased odds of exudative AMD occurrence (*p* = 0.033). Also, IL-10 serum levels were lower in exudative AMD than in controls (*p* = 0.049), patients with early AMD (*p* = 0.017), and atrophic AMD (*p* = 0.008). Furthermore, exudative AMD patients with *IL-10* rs1800896 CT and TT genotypes had lower IL-10 serum concentrations than those with wild-type (CC) genotype (*p* = 0.048). In conclusion, our study suggests that IL-10 serum levels can be associated with a minor allele at *IL-10* rs1800896 and exudative AMD. The haplotypes of IL-9 SNPs were also associated with the decreased odds of early and exudative AMD.

## 1. Introduction

Inflammation is a typical process involved in the pathogenesis of many diseases. While the inflammation is characterized as a signal transfer cascade which helps to identify and eliminate foreign materials and induce tissue recovery [1], the long-term inflammation and excessive proinflammatory molecule excretion can cause chronic conditions, such as cancer [2], type 2 diabetes mellitus [3], and neurodegenerative disorders [4], including age-related macular degeneration (AMD) [5]. AMD is a worldwide leading cause of progressive and irreversible blindness affecting 1 out of 4 people older than 75 years in developed countries [6]. Still, early signs of the disease can appear when people are in their 50s [7]. The particular pathophysiology of AMD is not clear, so this ocular impairment is described as a multifactorial disease because of its associations with environmental [8, 9] and genetic factors [10], metabolite profile [11], and even microbiome [12] changes. Increasing age, female gender, and ethnicity with the highest prevalence in Europeans at 12.3–30% have also been pinpointed as relevant risk factors [13, 14].

According to the Age-Related Eye Disease Study (AREDS), AMD is divided into early, intermediate, and late stages [15]. Early AMD is usually asymptomatic with defined lipid, protein, and collagen detachments between retinal pigment epithelium (RPE) and Bruch's membrane (BrM) in the retina [16] called drusen and retinal pigment abnormalities. The intermediate stage is described as a presence of at least one large drusen, numerous medium-sized drusen, or geographic atrophy (GA) without extension to the center of the macula. The late AMD is divided into dry or atrophic AMD with the GA of the RPE, and neovascular or exudative AMD is diagnosed when choroidal neovascularization with detachments in the RPE, hemorrhages, and/or scars appears and causes central vision impairments [17].

Drusogenesis or accumulation of lipids and other metabolites remains a significant AMD process, resulting in chronic inflammation that directly affects RPE, choroidal capillaries, and BrM [18]. The oxidative stress caused by reactive oxygen species (ROS), nitric oxide (NO), oxidized lipoproteins, advanced glycosylation end products (AGER), and apoptotic cells is the leading cause of ocular inflammation [19–21]. These accumulated substances also promote the RPE to release large amounts of different inflammatory factors. These factors' long-term exposure leads to the degeneration and atrophy of photoreceptors and RPE cells in the retina [22]. During the inflammation, complement system components are activated whose persistent accumulation impairs RPE and promotes inflammatory cells' (leukocytes, microglial cells, and macrophages) activation [18]; when macrophage recruitment is impaired at the site of inflammation, accumulating metabolites stimulate the release of proangiogenic mediators, such as vascular endothelial growth factor (VEGF), which induce progressive angiogenesis [19, 23–25]. New, however weak, permeable, and leaking blood vessels in the choroid cause local edema leading to acute vision loss with hemorrhages and fibrotic scars [26]. These pathological processes are responsible for the degradation of BrM and the extracellular matrix and lead to exudative AMD development [24]. Lin et al. found inflammation factors (IL-10, IL-1ra, IL-9, and IL-13) that may be associated with AMD's pathogenesis and revealed their function and regulation via specific NF-jB and JAK-STAT pathways, encouraging for the new exudative AMD treatment [23].

Genetic variations in cytokine coding genes can also cause cytokine expression changes that affect the balance between pro- and anti-inflammatory cytokines and disturb the appropriate immune response, leading to disease development. IL-10 level changes were linked to the genetic alterations mostly known as three *IL-10* -1082 (rs1800896), -819 (rs1800871), and -592 (rs1800872) promoter site single-nucleotide polymorphisms (SNPs) [27]. Otherwise, only one study reported SNP in the IL-9 gene associated with the IL-9 expression. It showed that individuals carrying the A allele of the -351 polymorphism in IL-9 promoter were linked to the increased synthesis of IL-9 [28].

Considering the immunological impairment in AMD development, we aimed to determine the possible associations of *IL-9* rs1859430, rs2069870, rs11741137, rs2069885, and rs2069884 and *IL-10* rs1800871, rs1800872, and rs1800896 polymorphisms and their haplotypes, as well as the serum levels of IL-9 and IL-10 with the early, exudative, and atrophic AMD. We also aimed to evaluate the associations between these polymorphisms and IL-9 and IL-10 concentrations.

## 2. Materials and Methods

### 2.1. Study Subjects

The study was approved by the Ethics Committee for Biomedical Research, Lithuanian University of Health Sciences (No. BE-2-/48).

The study groups were made of subjects who were admitted to the Hospital of Lithuanian University of Health Sciences Ophthalmology Department for preventive ophthalmological evaluation. In our study, 1209 participants were enrolled: 343 subjects in early AMD, 422 in exudative AMD, and 61 in the atrophic AMD groups. Also, 383 persons were involved as healthy controls (Table 1). Using the global AMD prevalence (8.7%) [13] and the minor allele frequencies from https://www.ncbi.nlm.nih.gov/snp/, we calculated that our collected sample sizes for the early and exudative AMD and control groups were sufficient to reach 80% or higher power for the selected SNP analysis. Unfortunately, the atrophic AMD group is too small to reach at least 80% power, and according to the power calculator (http://csg.sph.umich.edu/abecasis/cats/gas_power_calculator/), the sample size should be about 100 cases, but the atrophic AMD is a rarer condition than the early and exudative AMD in Lithuanian population to collect enough samples.

Study subjects underwent ophthalmological evaluation and general examination [29]. Participants were enrolled in our study, according to the previously published criteria [29].

### 2.2. Ophthalmological Evaluation

All the study subjects were evaluated by slit-lamp biomicroscopy to assess corneal and lenticular transparency. Classification and grading of lens opacities were performed according to the Lens Opacities Classification System III. On each examination, intraocular pressure was measured. Pupils were dilated with tropicamide 1%. Fundoscopy, using a direct monocular ophthalmoscope, and slit-lamp biomicroscopy with a double aspheric lens +78 diopters were performed. Results of eye examinations were recorded on most standardized forms. For a detailed analysis of the macula, stereoscopic color fundus photographs of the macula, centered at 45° and 30° to the fovea, were obtained with a Visucam NM Digital camera (Carl Zeiss Meditec AG, Germany).

All the AMD patients underwent optical coherence tomography (OCT), and fluorescence angiography was performed in patients suspected of having late AMD after the OCT examination.

The classification system of AMD formulated by the Age-Related Eye Disease Study was used: early mild AMD consisted of a combination of multiple small drusen and several intermediate (63–124 *μ*m in diameter) drusen, or retinal pigment epithelial abnormalities and the presence of extensive intermediate drusen characterized early intermediate AMD and at least one large (≥125 *μ*m in diameter) drusen, or geographic atrophy not involving the center of the fovea. Advanced AMD was characterized by geographic atrophy involving the fovea and/or any of the neovascular AMD features [15].

### 2.3. Control Group Formation

The control group consisted of subjects who had no ophthalmologic pathology on examination and agreed to participate in this study. After senile cataract surgeries, the patients were also included in the control group, while they have no other ocular comorbidity. The exclusion criteria were (i) unrelated eye disorders, e.g., high refractive error, cloudy cornea, lens opacity (nuclear, cortical, or posterior subcapsular cataract) except minor opacities, keratitis, acute or chronic uveitis, glaucoma, or diseases of the optic nerve; (ii) systemic illnesses, e.g., diabetes mellitus, malignant tumors, systemic connective tissue disorders, chronic infectious diseases, or conditions following organ or tissue transplantation; and (iii) ungraded color fundus photographs resulting from obscuration of the ocular optic system or because of fundus photograph quality.

### 2.4. General Medical Examination

Data on hypertension, diabetes mellitus, hyperlipidemia, coronary artery disease, and stroke were obtained during an examination by a family doctor and gathered from medical records for all study subjects.

### 2.5. Polymorphism Selection

As the IL-9 and IL-10 have been shown having interactions (https://version-11-0b.string-db.org/cgi/network?networkId=bMDXv9BDi65x), five SNPs (rs1859430, rs2069870, rs11741137, rs2069885, and rs2069884) in the IL-9 gene and three SNPs (rs1800871, rs1800872, and rs1800896) in the *IL-10* gene whose minor allele frequencies in the Europe population are more than 0.1 were selected from the dbSNP database https://www.ncbi.nlm.nih.gov/snp/. The rs2069885 is a missense variant (resulting in a threonine to methionine amino acid substitution), the rs1859430 and rs2069884 intronic variants are located in the coding region of *IL-9*, and rs11741137 is a downstream, and rs2069870 is an upstream gene variant with no known function. Moreover, rs1859430, rs11741137, rs2069884, and rs2069885 have already been published and identified as potential biomarkers for the other diseases [30–35]; also, rs2069870 along with the SNP in *IL-26* demonstrated influenced susceptibility to develop allergic rhinitis [36].

The rs1800871, rs1800872, and rs1800896 SNPs are located in the upstream *IL-10* promoter region and associated with transcription of IL-10 mRNA and IL-10 protein expression in vitro [37].

All these SNPs were selected for the association study in the AMD group for the first time, suggesting their role in IL-9 and IL-10 signaling pathway and AMD development.

### 2.6. The DNA Extraction and Genotyping

After collecting the venous blood samples, the DNA salting-out method was used for preparing genomic DNA from the white blood cells. Eight SNPs were genotyped on the *Step One Plus* real-time PCR system (Applied Biosystems, Foster City, USA). The TaqMan SNP genotyping assays for all eight chosen SNPs were performed according to the manufacturer's protocol.

For quality control, 5% of randomly chosen samples for each of the 8 SNPs were selected for repetitive analysis.

### 2.7. Quantification of IL-10 and IL-9 Serum Levels

IL-9 and IL-10 serum levels were measured in 19 control subjects, 20 patients with early AMD, 20 atrophic AMD, and 26 exudative AMD. These two assays were performed using the Invitrogen ELISA Kit (Cat. No. BMS2081) for human IL-9, standard curve sensibility range: 3.1–200 pg/mL, sensitivity 0.5 pg/mL; Invitrogen ELISA Kit (Cat. No. KHC0101) for human IL-10, standard curve sensibility range: 0–500 pg/mL, sensitivity <1 pg/mL, following the manufacturer's instructions, and they were analyzed on the Multiskan FC Microplate Photometer (Thermo Scientific, Waltham, MA) at 450 nm. The samples were excluded if the levels of serum cytokines were below the detection range.

### 2.8. Statistical Analysis

Statistical analysis was performed using the SPSS/W 20.0 software (Statistical Package for the Social Sciences for Windows, Inc., Chicago, Illinois, USA). Age and interleukin serum level data distributions were evaluated for normality by the Kolmogorov-Smirnov test. Continuous variables presented median with interquartile range (IQR) based on data distribution. For nonnormally distributed data, Mann-Whitney *U* test was used to compare two groups, and the Kruskal-Wallis test for the three groups (statistically significant differences observed when *p* < 0.05).

Categorical data (gender, genotype, and allele distributions) are presented as absolute numbers with percentages in brackets and compared between the early, exudative, and atrophic AMD and control groups using the *χ*^2^ test.

The impact of gene polymorphisms on early, exudative, and atrophic AMD was evaluated using binomial logistic regression analysis and presented as odds ratios (ORs) with 95% confidence interval (CI) after gender adjustment in early AMD and age in the exudative and atrophic AMD groups. Logistic regression analysis results were expressed as genetic models (codominant: heterozygotes versus wild-type homozygotes and minor allele homozygotes versus wild-type homozygotes; dominant: minor allele homozygotes and heterozygotes versus wild-type homozygotes; recessive: minor allele homozygotes versus wild-type homozygotes and heterozygotes; overdominant: heterozygotes versus wild-type homozygotes and minor allele homozygotes; the additive model was used to evaluate the impact of each minor allele on AMD). The best genetic model selection was based on the Akaike information criterion (AIC); therefore, the best genetic models were those with the lowest AIC values. We introduced an adjusted significance threshold for multiple comparisons *α* = 0.00625 (0.05/8, since we analyzed eight different SNPs) due to multiple association calculations [38].

Haplotype analysis was performed between the early AMD and control groups, exudative AMD and control groups, and atrophic AMD and control groups using online SNPStats software (https://www.snpstats.net/snpstats/) [39]. Two haplotype blocks were constructed based on different chromosomes where the SNPs were located. Linkage disequilibrium (LD) analysis was assessed by *D*′ and *r*^2^ measures. The associations between the haplotypes with frequencies of at least 1% and different AMD forms were calculated by logistic regression and presented as ORs and 95% CI and *p* values adjusted for gender in early AMD and age in exudative and atrophic AMD analysis. Statistically significant differences observed when *p* < 0.05.

## 3. Results

### 3.1. Hardy-Weinberg Equilibrium

We evaluated the distributions of rs1859430, rs2069870, rs11741137, rs2069885, rs2069884, rs1800871, rs1800872, and rs1800896 genotypes in the control group using the Hardy-Weinberg equilibrium (HWE). Seven SNPs were in HWE (*p* > 0.05), but rs2069870 did not fulfill the HWE requirements because there were observed only two genotypes (Supplementary Materials, Table S1).

### 3.2. *IL-9* (rs1859430, rs2069870, rs11741137, rs2069885, and rs2069884) and *IL-10* (rs1800871, rs1800872, and rs1800896) Analysis in Early, Exudative, and Atrophic AMD

We analyzed 8 SNPS (rs1859430, rs2069870, rs11741137, rs2069885, rs2069884, rs1800871, rs1800872, and rs1800896) and their genotype and allele distributions between the early, exudative, and atrophic AMD and control groups. Our statistical analysis revealed that genotype distributions of *IL-9* rs1859430 (GG, GA, and AA) differ between the early AMD and control groups (65.6%, 27.7%, and 6.7% vs. 60.8%, 35.5%, and 3.7%, *p* = 0.024). Any differences between study groups and *IL-9* (rs2069870, rs11741137, rs2069885, and rs2069884) and *IL-10* (rs1800871, rs1800872, and rs1800896) were found (Table 2).

We performed the binary logistic regression analysis to evaluate these SNPs' impacts on early, exudative, and atrophic AMD. The analysis showed that *IL-9* rs1859430 GA genotype was associated with 30% decreased odds of early AMD (OR = 0.700; CI: 0.507-0.966; *p* = 0.030) under the codominant model, and about 33% decreased under the overdominant model after adjustment for gender (OR = 0.673; CI: 0.490-0.925; *p* = 0.015). *IL-9* rs11741137 CT genotype was associated with 28% decreased odds of early AMD under the overdominant model after adjustment for gender (OR = 0.720; CI: 0.522-0.994; *p* = 0.046). Also, we found that *IL-10* rs1800896 CC genotype was associated with 2-fold increased odds of atrophic AMD (OR = 2.013; CI: 1.078-3.759; *p* = 0.028) under the recessive model after adjustment for age (Table 3). Since we analyzed 8 SNPs in our study, we applied the Bonferroni correction (significance threshold, *p* = 0.05/8), and the results did not survive this strict correction. No statistically significant associations were found in the exudative AMD group (data not shown).

While it has been suggested that AMD pathogenesis can be differentiated by gender [14], we performed the SNP analysis in males and females separately and found that *IL-9* rs11741137 (CC, CT, and TT), *IL-9* rs2069885 (GG, GA, and AA), and *IL-9* rs2069884 (GG, GT, and TT) genotypes were distributed statistically significantly between males with early AMD and control males: 73.3%, 20%, and 6.7 vs. 66.4%, 31.5%, and 2%, *p* = 0.032; 73.3%, 20%, and 6.7 vs. 67.1%, 30.9%, and 2%, *p* = 0.039; 73.3%, 20%, and 6.7 vs. 67.1%, 30.9%, and 2%, *p* = 0.039, respectively (Table 4). No statistically significant associations were found in the exudative or atrophic AMD groups and female group analysis (data not shown).

Binomial logistic regression analysis revealed that *IL-9* rs1859430 GA genotype was associated with 46 and 48% decreased odds of early AMD in males under the codominant and overdominant genetic models (OR = 0.547; CI: 0.302-0.991; *p* = 0.047 and OR = 0.526; CI: 0.292-0.948; *p* = 0.033, respectively). *IL-9* rs11741137 CT genotype was associated with 46% decreased odds of early AMD in males (OR = 0.543; CI: 0.301-0.979; *p* = 0.042) under the codominant model (Table 5).

None of the results survived strict Bonferroni correction (significance threshold, *p* = 0.05/8).

### 3.3. Haplotype Association with the Predisposition to AMD Occurrence

Strong linkage disequilibrium between studied polymorphisms was observed (Table 6).

While the haplotype analyses identified many of their sets, any differences in the haplotype frequencies between the atrophic AMD and control groups were observed (Table 7). The results of the frequencies of haplotypes among patients with early AMD and controls have shown that haplotypes A-G-C-G-G and G-A-T-A-T of *IL-9* SNPs (rs1859430, rs2069870, rs11741137, rs2069885, and rs2069884) are associated with the decreased odds of early AMD occurrence (OR = 0.49; 95% CI: 0.025-0.95; *p* = 0.035 and OR = 0.08; 95% CI: 0.01-0.61; *p* = 0.015, respectively). The set of rare haplotypes was associated with the decreased odds of exudative AMD occurrence (OR = 0.37; 95% CI: 0.015-092; *p* = 0.033) (Table 7).

### 3.4. IL-9 and IL-10 Serum Levels in the AMD and Control Groups

**Figure 1:**
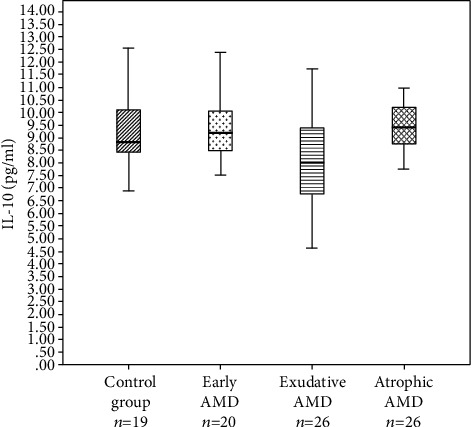
Interleukin 10 concentrations between the groups. IL-10 serum levels in the control (8.8 pg/ml), early AMD (9.2 pg/ml), exudative AMD (8.0 pg/ml), and atrophic AMD (9.4 pg/ml) groups. Kruskal-Wallis test (*p* = 0.020). The bars represent the median with interquartile variation (1^st^ quartile and 3^rd^ quartile).

IL-9 and IL-10 serum levels were measured in patients with early AMD (*n* = 20), exudative AMD (*n* = 26), atrophic AMD (*n* = 20), and controls (*n* = 19). Subgroups for interleukin serum level measurements consisted of study subjects considering the age and gender distributions in subgroups. IL-9 levels did not reach the detection range, and it was not analyzed. IL-10 serum levels differed between study groups (*p* = 0.02) (Figure 1). When comparing the IL-10 serum levels between every two groups, we found that IL-10 serum levels were lower in exudative AMD than in controls (8.0 (2.7) pg/ml vs. 8.8 (2.4) pg/ml, *p* = 0.049) and also in patients with early AMD (8.0 (2.7) pg/ml vs. 9.2 (1.7) pg/ml, *p* = 0.017) and atrophic AMD (8.0 pg/ml vs. 9.4 (1.5) pg/ml, *p* = 0.008).

We also performed the IL-10 serum level and SNP association analysis and found that exudative AMD patients with *IL-10* rs1800896 CT and TT genotypes had lower IL-10 serum concentrations than those with wild-type (CC) genotype: 7.2 (2.3) *vs.* 9.3 (1.4); *p* = 0.048 (Table 8).

## 4. Discussion

Our study is aimed at analyzing the associations between the immunogenetic markers *IL-9* (rs1859430, rs2069870, rs11741137, rs2069885, and rs2069884) and *IL-10* (rs1800871, rs1800872, and rs1800896) polymorphisms and their haplotypes, serum IL-9 and IL-10 levels and the different AMD forms.

IL-9 belongs to the IL-2R*γ*c chain family and works as a pleiotropic cytokine in inflammatory processes [40]. T lymphocytes, or more specific Th2, were described as the primary source for the IL-9 production [41]. Nevertheless, further studies identified other cell types, including Th9, mast cells, innate lymphoid cells (ILCs), NK cells, and even Foxp3+ Tregs, as well as mucin-producing cells, and eosinophils could also produce IL-9 [42–44]. Moreover, Dardalhon et al. have identified unique T cells that produce both IL-9 and IL-10, leading to tissue inflammation [45]. Previously, IL-9 was described as a growth factor for T cells and mast cells [46, 47]. It is known that IL-9 can promote the growth and function of the erythroid progenitor, fetal thymocyte, myeloid precursor cells, and human megakaryoblastic leukemic cell lines [48]. IL-9 behavior is regulated through the specific IL-9 receptor (IL9R, which is contained of two subunits: the alpha chain (IL-9R*α*) and the common gamma chain receptor). IL-9 binds the IL-9R*α* subunit and forms the IL-9R heterocomplex. Because of the lack of specific enzymatic activity, the JAK/STAT pathway needs to be activated, and JAK is the initiator of the following phosphorylation cascades [49]. Previous inflammation-associated studies were reviewed and showed the pathogenic role of IL-9 in inflammatory disease development [48]. On the other hand, Elyaman et al. have been suggested the diverse role of IL-9, including both a regulator of pathogenic and protective mechanisms of immune responses [50].

IL-9 is encoded by the *IL-9* gene located on chromosome 5q31.1 [51]. Several *IL-9* variants (rs31563, rs1859430, rs11741137, and rs2069885) have already been published and identified as potential biomarkers for atopic dermatitis, asthma and its severity, respiratory syncytial virus (RSV) infection, and pituitary adenoma [30–35].

Namkung et al. reported that rs31563 (−4091G/A) at the *IL-9* gene was associated with increased susceptibility to atopic dermatitis [34]. Another study revealed that *IL-9* rs1859430 genotype frequencies were lower in asthma patients than in controls under the recessive GA+AA (*p* = 0.021) and heterozygous GA (*p* = 0.031) models. Also, those patients had significantly lower A-T (rs1859430-rs2066758) haplotype frequency (*p* = 0.006) and higher G-T (rs1859430-rs2066758) haplotype frequency (*p* ≤ 0.001). Moreover, they showed that rs1859430 AG genotype was associated with the higher IL-9 serum levels compared with other genotypes in the disease group (*p* < 0.05), and the rs2066758 CC genotype was linked to the partial pressure of carbon dioxide (PaCO_2_) (*p* = 0.041) [35]. Two authors underlined the differences between gender groups in asthma and RSV infection development, considering the sex-dependent mechanisms. Aschard et al. showed that polysensitization (SPTQ) and forced expiratory volume in one second divided by height square (FEV(1)/H(2)) were associated with two *IL-9* variants rs2069885 and rs2069882 (*p* = 0.02 and *p* = 0.002, respectively, after Bonferroni's correction). This study underlines the importance of complex mechanisms, such as heterogeneity, according to sex and pleiotropy, to reveal the genes involved in asthma phenotypes [33]. Schuurhof et al. revealed that the major allele at rs2069885 was associated with increased susceptibility to severe RSV infection in boys' and girls' opposite associations. Furthermore, the haplotype T-T rs2069885 and rs1799962 was a risk marker for severe RSV bronchiolitis in girls [32]. One more study found that study subjects with the dominant genotype for these *IL-9* polymorphisms (rs11741137, rs2069885, and rs1859430) were associated with a severe asthma exacerbation if exposed to increased dust mite levels (*p* = 0.02 to 0.03). It was even replicated it in another study (*p* = 0.04) [52]. *IL-9* rs1859430 G/A and A/A genotypes were also found to be associated with increased odds of having recurrent PA under the codominant (*p* = 0.003 and *p* = 0.006, respectively), dominant (*p* = 0.011), and recessive (*p* = 0.021) genetic models [30].

Controversial results were observed in several studies as well, and they reported no significant associations between *IL-9* promoter polymorphism A−345G and RSV bronchiolitis [52], T113M in *IL-9* and atopic bronchial asthma [53], rs1859430 and rs2069868 and Graves' disease [54], or *IL-9* rs2069885 and allergic rhinitis in Iranian women [55]. Schürks et al. tried to show associations between *IL-9* rs2069885 and inflammatory pathways among women with migraine, but results did not survive the corrections for multiple testing [56]. Also, *IL-9* rs31563 C>T and rs31564 G>T were not associated with gastric cardiac adenocarcinoma and esophageal cancer in a Chinese population [57, 58].

According to the databases, any *IL-9* SNPs were analyzed in AMD. Our study shows that *IL-9* rs1859430 GA genotype and *IL-9* rs11741137 CT genotypes were associated with decreased odds of early AMD. Unfortunately, in our study, we applied Bonferroni's correction because of the multiple comparisons, and associations between study groups and SNPs in *IL-9* did not survive this strict correction.

In further analysis, the same tendencies remained only in the male group. Even if these results did not survive Bonferroni's correction, differences between male and female groups were observed as in previous studies [14, 32, 33]. Potentially different inflammation processes in men and women should be studied, considering the hormone role on molecular mechanisms in inflammation and response to inflammation, which may lead to opposite disease outcomes [59].

Also, we performed the haplotype analysis and identified the haplotypes A-G-C-G-G and G-A-T-A-T of *IL-9* SNPs (rs1859430, rs2069870, rs11741137, rs2069885, and rs2069884), which were associated with the decreased odds of early AMD (*p* = 0.035 and *p* = 0.015, respectively). The set of rare haplotypes was associated with the decreased odds of exudative AMD (*p* = 0.033), which may have a potential role in the IL-9-dependent inflammation process involved in AMD development.

In our study, the serum IL-9 levels were not determined because of the low detection rates. On the other hand, IL-9 was previously involved in several AMD studies. IL-9 cytokine in the aqueous humor was measured, but differences between neovascular AMD and the control groups were not found [60]. IL-9 was also measured in aqueous humor samples of neovascular AMD before the treatment by intravitreal drug injection and after, and compared with the control group, but the statistical analysis did not show any differences between study groups [61]. In another study, plasma and aqueous humor IL-9 levels between exudative AMD and the control groups were not reported because of the low detection rate [62]. Extremely low limits of detection of IL-9 in the aqueous fluid of patients with neovascular AMD were reported as well [63].

Contrarily, Lin et al. revealed that cytokine IL-9 was overexpressed in stimulated RPE cells, with a potential association with AMD development [23]. Unfortunately, no other studies, including IL-9 and AMD, were found. Otherwise, few diseases/conditions were reported, and elevated IL-9 levels were found in asthmatic patients, patients with allergic rhinitis and a peanut allergy, and those suffering from some autoimmune diseases [48].

IL-10 belongs to the IL-10 family, and as an anti-inflammatory cytokine, the IL-10 takes part in the regulation of the inflammatory response [64]. Macrophages are the primary source of IL-10. Still, other immune cells (monocytes, dendritic cells, B lymphocytes, T helper 1 (Th1) and Th2 lymphocytes, mast cells, NK cells, cytotoxic T cells, and granulocytes like neutrophils and eosinophils) can secrete this interleukin [65, 66]. As a pleiotropic cytokine, IL-10 inhibits the antigen-presenting cells via the inhibition of expression of major histocompatibility complex (MHC) class II molecules. It also suppresses the expression of IL-1, IL-6, IL-8, IL-12, and tumor necrosis factor-alpha (TNF-*α*). Furthermore, IL-10 promotes proliferation, activation, and differentiation and helps prevent cell apoptosis in B cells [27, 65]. The immunosuppressive IL-10 activity is mediated by the heterodimeric IL-10 receptors (IL-10R1 and IL-10R2). IL-10R1 primary ligates to IL-10 and dimerizes with IL10R2 leading to the activation of the Janus kinase/signal transducer and activator of transcription (JAK/STAT) signaling pathway. Phosphorylated STAT3 molecules that enter the cell's nucleus induce changes in the expression of immunomodulatory genes leading to the inhibition of the secreted proinflammatory cytokines and downstream immune response, and regulate the activity of growth factors, such as VEGF [67, 68]. The IL-10 level changes were reported as a significant pathophysiological modulator in many diseases and reviewed previously [27].

IL-10 and genetic variants of *IL-10* were also included in our study. IL-10 is encoded by the *IL-10* gene located on chromosome 1q32.1, and three most studied point mutations in *IL-10* promoter -592 A/C (rs1800872), -819 C/T (rs1800871), and -1082G/A (rs1800896) were described as leading genetic variations for SNP association studies [37]. Our present study showed that the *IL-10* rs1800896 CC genotype was associated with 2-fold increased odds of atrophic AMD (*p* = 0.028), but these results did not survive strict Bonferroni's correction as well.

Shevchenko et al. reported similar results. They found a higher *IL10*-1082 GG genotype frequency in AMD patients than in the controls [69], while in another study, the associations of *IL-10* -592 A/C, -819 C/T, and -1082G/A polymorphisms and late AMD were not determined [70]. Moreover, no more such studies were found, but these SNPs were associated with other conditions. For example, children with *IL-10* -592 CC or -592 AA genotypes had a higher risk of hospitalization for RSV bronchiolitis than those with heterozygous genotype [52]. Also, *IL-10* rs1800872 T>G polymorphism was associated with an increased risk of esophageal cancer in a Chinese population [58]. It was still not associated with gastric cardiac adenocarcinoma in the same population [57], suggesting further investigations for the IL-10 signaling pathway associations with cancer development.

Previous studies have also shown that the elevated IL-10 levels in the eye induce the alternative macrophage activation, which can be associated with choroidal neovascularization development [71, 72]. We also analyzed serum IL-10 levels and found that IL-10 serum levels were lower in exudative AMD than in controls (*p* = 0.049), and also in patients with early AMD (*p* = 0.017) and atrophic AMD (*p* = 0.008). Similar results were found by the other researcher group, which revealed lower concentrations of IL-10 cytokine in the wet and dry AMD groups than in controls (*p* < 0.05 and *p* < 0.05). Also, they determined that IL-10 levels were higher in wet AMD than in the dry AMD group (*p* = 0.009) [73]. Opposite results were found when Subhi et al. revealed that patients with neovascular AMD had higher plasma levels of IL-10 compared to healthy controls (*p* < 0.0001), and statistically significant results remained even after multivariate analysis (after adjusting for demographics, comorbidities, and lifestyle factors) IL-10 (*p* < 0.001) [74]. Statistically, significantly elevated IL-10 serum levels in AMD patients were determined by Nassar et al. as well [75]. On the other hand, the expression of IL-10 did not differ between Tfh cells from AMD patients and non-AMD controls [76]. It is interesting that IL-10 levels in the aqueous humor did not differ between the neovascular AMD and control groups [61–63]. Few other studies have not even determined IL-10 in plasma or aqueous humor samples of AMD patients [61–63]. In contrast, the others showed that IL-10 levels do not differ between intraocular fluid and serum samples [77].

Moreover, we found that IL-10 levels in the exudative AMD group are associated with the minor allele T, and patients with exudative AMD carrying *IL-10* rs1800896 CT and TT genotypes have lower IL-10 serum concentrations than those with wild-type (CC) genotype. These findings confirm the *IL-10* promoter polymorphism (rs1800896) role on IL-10 level changes [37], which can be responsible for the immune response in exudative AMD development. It is important to highlight that *IL-9* (rs1859430, rs2069870, rs11741137, rs2069885, and rs2069884) and *IL-10* (rs1800871, rs1800872, and rs1800896) gene variants, as well as serum IL-9 and IL-10 levels, have never been studied in AMD, in the Lithuanian population, and our study was the first of its type. While a thorough medical examination of the study objects can be acknowledged as one of our study's main strengths, we should declare that the relatively small sample size and the other risk factors that were not involved in our study fall into this study's limitations.

## 5. Conclusions

In conclusion, inflammation is an underlying mechanism in AMD development. We have found lower IL-10 serum levels in patients with exudative AMD than healthy controls and early or atrophic AMD patients. A minor allele at *IL-10* rs1800896 was associated with the lower IL-10 serum levels in the exudative AMD group. The haplotypes of IL-9 SNPs were also associated with the decreased odds of early and exudative AMD occurrence. Further studies will be needed to elucidate this regulatory pathway's underlying mechanism and its association with AMD clinical symptoms.

## Figures and Tables

**Table 1:** Demographic data of the studied groups.

	Early AMD group (*n* = 343)	Exudative AMD group (*n* = 422)	Atrophic AMD group (*n* = 61)	Control group (*n* = 383)	*p* value
Males, *n* (%)	105 (30.6)	149 (35.3)	22 (36.1)	149 (38.9)	0.019^∗^ 0.291^∗∗^ 0.672^∗∗∗^
Females, *n* (%)	238 (69.4)	273 (64.7)	39 (63.9)	234 (61.1)	
Age, median (IQR)	73 (13)	77 (10)	80 (9)	72 (11)	0.266^∗^ <0.001^∗∗^ <0.001^∗∗∗^

^∗^Early AMD vs. control group. ^∗∗^Exudative AMD vs. control group. ^∗∗∗^Atrophic AMD vs. control group. IQR: interquartile range; *p*: significance level, statistically significant differences observed when *p* < 0.05.

**Table 2:** Distributions of IL-9 and IL-10 SNP genotypes and alleles in the early, exudative, and atrophic AMD and control groups.

SNP	Genotypes/alleles	Group				*p* value^∗^	*p* value^∗∗^	*p* value^∗∗∗^
		Early AMD (*n* = 343), *n* (%)	Exudative AMD (*n* = 422), *n* (%)	Atrophic AMD (*n* = 61), *n* (%)	Control (*n* = 383), *n* (%)			
*IL-9* rs1859430	GG	225 (65.6)	262 (62.1)	33 (54.1)	233 (60.8)	0.024	0.871	0.426
	GA	95 (27.7)	143 (33.9)	24 (39.3)	136 (35.5)			
	AA	23 (6.7)	17 (4.0)	4 (6.6)	14 (3.7)			
	G	545 (79.4)	667 (79.0)	90 (73.8)	602 (78.6)	0.625	0.472	0.088
	A	141 (20.6)	177 (21.0)	32 (26.2)	146 (21.4)			

*IL-9* rs2069870	AA	222 (64.7)	262 (62.1)	33 (54.1)	235 (61.4)	0.349	0.832	0.282
	AG	121 (35.3)	160 (37.9)	28 (45.9)	148 (38.6)			
	GG	0 (0)	0 (0)	0 (0)	0 (0)			
	A	565 (82.4)	684 (81.0)	94 (77.0)	618 (80.7)	0.410	0.853	0.350
	G	121 (17.6)	160 (19.0)	28 (23.0)	148 (19.3)			

*IL-9* rs11741137	CC	238 (69.4)	290 (68.7)	41 (67.2)	247 (64.5)	0.117	0.389	0.846
	CT	91 (26.5)	120 (28.6)	18 (29.5)	126 (32.9)			
	TT	14 (4.1)	12 (2.8)	2 (3.3)	10 (2.6)			
	C	657 (82.7)	700 (82.9)	100 (82.0)	620 (80.9)	0.053	0.297	0.788
	T	119 (17.3)	144 (17.1)	22 (18.0)	146 (19.1)			

*IL-9* rs2069885	GG	240 (70.0)	293 (69.4)	41 (67.2)	250 (65.3)	0.172	0.453	0.892
	GA	90 (26.2)	119 (28.2)	18 (29.5)	123 (32.1)			
	AA	13 (3.8)	10 (2.4)	2 (3.3)	10 (2.6)			
	G	570 (83.1)	705 (83.5)	100 (82.0)	623 (81.3)	0.382	0.24	60.867
	A	116 (16.9)	139 (16.5)	22 (18.0)	143 (18.7)			

*IL-9* rs2069884	GG	239 (69.7)	292 (69.2)	41 (67.2)	250 (65.3)	0.199	0.495	0.892
	GT	91 (26.5)	120 (28.4)	18 (29.5)	123 (32.1)			
	TT	13 (3.8)	10 (2.4)	2 (3.3)	10 (2.6)			
	G	569 (82.9)	704 (83.4)	100 (82.0)	623 (81.3)	0.424	0.273	0.867
	T	117 (17.1)	140 (16.6)	22 (18.0)	143 (18.7)			

*IL-10* rs1800871	GG	208 (60.6)	252 (59.7)	38 (62.3)	232 (60.6)	0.705	0.903	0.957
	GA	123 (35.9)	152 (36.0)	20 (32.8)	133 (34.7)			
	AA	12 (3.5)	18 (4.3)	3 (4.9)	18 (4.7)			
	G	539 (78.6)	656 (77.7)	96 (78.7)	597 (77.9)	0.770	0.918	0.852
	A	147 (21.4)	188 (22.3)	26 (21.3)	169 (22.1)			

*IL-10* rs1800872	GG	208 (60.6)	252 (59.7)	38 (62.3)	232 (60.6)	0.705	0.903	0.957
	GT	123 (35.9)	152 (36.0)	20 (32.8)	133 (34.7)			
	TT	12 (3.5)	18 (4.3)	3 (4.9)	18 (4.7)			
	G	539 (78.6)	656 (77.7)	96 (78.7)	597 (77.9)	0.770	0.918	0.852
	T	147 (21.4)	188 (22.3)	26 (21.3)	169 (22.1)			

*IL-10* rs1800896	TT	103 (30.0)	112 (26.5)	14 (23.0)	103 (26.9)	0.642	0.319	0.084
	TC	175 (51.0)	207 (49.1)	27 (44.3)	203 (53.0)			
	CC	65 (19.0)	103 (24.4)	20 (32.8)	77 (20.1)			
	T	381 (55.5)	431 (51.1)	55 (45.1)	409 (53.4)			
	C	305 (44.5)	413 (48.9)	67 (54.9)	357 (46.6)	0.413	0.350	0.088

^∗^Early AMD vs. control group. ^∗∗^Exudative AMD vs. control group. ^∗∗∗^Atrophic AMD vs. control group. *p*: significance level and Bonferroni corrected significance level when *p* = 0.05/8.

**Table 3:** The impact of IL-9 rs185943 and rs11741137 on early AMD and IL-10 rs1800896 on atrophic AMD.

Model	Genotype/allele	OR^∗^ (95% CI)	*p* value	AIC
*Early AMD*				
*IL-9* rs1859430				
Codominant	GA vs. GG	0.700 (0.507-0.966)	**0.030**	1000.541
	AA vs. GG	1.713 (0.857-3.424)	0.128	
Dominant	GA+AA vs. GG	0.794 (0.585-1.077)	0.137	998.541
Recessive	AA vs. GA+GG	1.926 (0.972-3.816)	0.060	997.109
Overdominant	GA vs. GG+AA	0.673 (0.490-0.925)	**0.015**	994.759
Additive	A	0.938 (0.731-1.203)	0.613	1000.499
*IL-9* rs11741137				
Codominant	CT vs. CC	0.734 (0.530-1.015)	0.062	997.863
	TT vs. CC	1.489 (0.646-3.431)	0.350	
Dominant	CT+TT vs. CC	0.788 (0.577-1.077)	0.135	998.512
Recessive	TT vs. CT+CC	1.635 (0.714-3.745)	0.245	999.381
Overdominant	CT vs. CC+TT	0.720 (0.522-0.994)	**0.046**	996.749
Additive	T	0.883 (0.675-1.156)	0.365	999.931

*Atrophic AMD*				
*IL-10* rs1800896				
Codominant	TC vs. TT	1.023 (0.503-2.079)	0.951	321.969
	CC vs. TT	2.043 (0.938-4.450)	0.072	
Dominant	TC+CC vs. TT	1.296 (0.670-2.505)	0.441	323.962
Recessive	CC vs. TC+TT	2.013 (1.078-3.759)	**0.028**	319.973
Overdominant	TC vs. TT+CC	0.719 (0.408-1.266)	0.253	323.257
Additive	C	1.454 (0.967-2.187)	0.072	321.302

^∗^Adjusted for gender in early AMD and adjusted for age in atrophic AMD group. OR: odds ratio; CI: confidence interval; *p*: significance level and Bonferroni corrected significance level when *p* = 0.05/8; AIC: Akaike information criterion.

**Table 4:** Distributions of IL-9 rs11741137, rs2069885, and rs2069884 genotypes and alleles in the early AMD and control male groups.

SNP	Genotypes/alleles	Group		*p* value
		Early AMD (*n* = 105), *n* (%)	Control (*n* = 149), *n* (%)	
rs11741137	CC	77 (73.3)	99 (66.4)	**0.032**
	CT	21 (20.0)	47 (31.5)	
	TT	7 (6.7)	3 (2.0)	
	C	175 (83.3)	245 (82.2)	0.743
	T	35 (13.3)	53 (17.8)	

rs2069885	GG	77 (73.3)	100 (67.1)	**0.039**
	GA	21 (20.0)	46 (30.9)	
	AA	7 (6.7)	3 (2.0)	
	G	175 (83.3)	246 (82.6)	0.818
	A	35 (13.3)	52 (17.4)	

rs2069884	GG	77 (73.3)	100 (67.1)	**0.039**
	GT	21 (20.0)	46 (30.9)	
	TT	7 (6.7)	3 (2.0)	
	G	175 (83.3)	246 (82.6)	0.818
	T	35 (13.3)	52 (17.4)	

*p*: significance level and Bonferroni corrected significance level when *p* = 0.05/8.

**Table 5:** The impact of IL-9 rs1859430 and rs11741137 on early AMD in males.

Model	Genotype/allele	OR (95% CI)	*p* value	AIC
*IL-9* rs1859430				
Codominant	GA vs. GG	0.547 (0.302-0.991)	**0.047**	342.864
	AA vs. GG	1.667 (0.54-5.010)	0.363	
Dominant	GA+AA vs. GG	0.671 (0.390-1.155)	0.150	344.355
Recessive	AA vs. GA+GG	1.966 (0.661-5.843)	0.224	342.961
Overdominant	GA vs. GG+AA	0.526 (0.292-0.948)	**0.033**	341.702
Additive	A	0.863 (0.562-1.327)	1.327	346.005

*IL-9* rs11741137				
Codominant	CT vs. CC	0.574 (0.317-1.041)	0.068	341.517
	TT vs. CC	3.000 (0.751-11.983)	0.120	
Dominant	CT+TT vs. CC	0.720 (0.415-1.248)	0.242	345.071
Recessive	TT vs. CT+CC	3.476 (0.878-13.769)	0.076	342.969
Overdominant	CT vs. CC+TT	0.543 (0.301-0.979)	**0.042**	342.175
Additive	T	0.929 (0.590-1.464)	0.751	346.357

^∗^Only two genotypes were determined. OR: odds ratio; CI: confidence interval; p: significance level and Bonferroni corrected significance level when *p* = 0.05/8; AIC: Akaike information criterion.

**Table 6:** Linkage disequilibrium between studied polymorphisms.

	SNP1 (*D*′; *r*^2^)	SNP2 (*D*′; *r*^2^)	SNP3 (*D*′; *r*^2^)	SNP4 (*D*′; *r*^2^)	SNP5 (*D*′; *r*^2^)	SNP6 (*D*′; *r*^2^)	SNP7 (*D*′; *r*^2^)	SNP8 (*D*′; *r*^2^)
SNP1 (*D*′; *r*^2^)		0.9828; 0.8339	0.9167; 0.6752	0.9397; 0.6877	0.9401; 0.6922			
SNP2 (*D*′; *r*^2^)			0.8271; 0.6366	0.8436; 0.6419	0.8445; 0.6471			
SNP3 (*D*′; *r*^2^)				0.9942; 0.9579	0.9942; 0.9633			
SNP4 (*D*′; *r*^2^)					0.9995; 0.9932			
SNP5 (*D*′; *r*^2^)								
SNP6 (*D*′; *r*^2^)							0.9997; 0.9994	0.9883; 0.2454
SNP7 (*D*′; *r*^2^)								0.9883; 0.2454
SNP8 (*D*′; *r*^2^)								

*D*′ is the deviation between the expected haplotype frequency and the observed frequency [*D*′ scale: 0,1]. *r*^2^ is squared correlation coefficient of the haplotype frequencies [*r*^2^ scale: 0,1]. SNP1: rs1859430; SNP2: rs2069870; SNP3: rs11741137; SNP4: rs2069885; SNP5: rs2069884; SNP6: rs1800871; SNP7: rs1800872; SNP8: rs1800896.

**Table 7:** Haplotype association with the predisposition to AMD occurrence.

	SNP1	SNP2	SNP3	SNP4	SNP5	SNP6	SNP7	SNP8	Frequency	OR (95% CI)	*p* value
Haplotype associations with early AMD											
1	G	A	C	G	G	—	—	—	0.7715	1	—
2	A	G	T	A	T	—	—	—	0.1468	0.76 (0.54-1.08)	0.13
3	A	G	C	G	G	—	—	—	0.0335	0.49 (0.25-0.95)	**0.035**
4	A	A	T	A	T	—	—	—	0.018	1.74 (0.72-4.22)	0.22
5	G	A	T	A	T	—	—	—	0.0108	0.08 (0.01-0.61)	**0.015**
6	A	A	C	G	G	—	—	—	0.0103	2.25 (0.66-7.66)	0.19
7 (rare)	∗	∗	∗	∗	∗	—	—	—	0.009	1.29 (0.32-5.21)	0.72
8	—	—	—	—	—	G	G	C	0.4549	1	—
9	—	—	—	—	—	G	G	T	0.3275	1.13 (0.89-1.44)	0.31
10	—	—	—	—	—	A	T	T	0.2166	1.00 (0.75-1.32)	0.98
11 (rare)	—	—	—	—	—	∗	∗	∗	0.001	<0.001 (-)	1

Haplotype associations with exudative AMD											
1	G	A	C	G	G	—	—	—	0.7713	1	—
2	A	G	T	A	T	—	—	—	0.1487	0.96 (0.69-1.35)	0.83
3	A	G	C	G	G	—	—	—	0.0408	0.99 (0.57-1.73)	0.98
4	A	A	T	A	T	—	—	—	0.0165	1.11 (0.46-2.66)	0.82
5 (rare)	∗	∗	∗	∗	∗	—	—	—	0.0227	0.37 (0.15-0.92)	**0.033**
6	—	—	—	—	—	G	G	C	0.4751	1	—
7	—	—	—	—	—	G	G	T	0.3026	0.87 (0.68-1.10)	0.24
8	—	—	—	—	—	A	T	T	0.2205	0.94 (0.72-1.22)	0.63
9 (rare)	—	—	—	—	—	∗	∗	∗	0.0018	0.34 (0.02-5.66)	0.45

Haplotype associations with atrophic AMD											
1	G	A	C	G	G	—	—	—	0.7558	1	—
2	A	G	T	A	T	—	—	—	0.1518	1.25 (0.65-2.40)	0.5
3	A	G	C	G	G	—	—	—	0.0453	2.34 (0.93-5.88)	0.071
4	G	A	T	A	T	—	—	—	0.0167	<0.00 (-)	1
5	A	A	T	A	T	—	—	—	0.0162	1.05 (0.23-4.68)	0.95
6 (rare)	∗	∗	∗	∗	∗	—	—	—	0.0142	0.55 (0.07-4.43)	0.57
7	—	—	—	—	—	G	G	C	0.4758	1	—
8	—	—	—	—	—	G	G	T	0.3046	0.62 (0.38-1.02)	0.06
9	—	—	—	—	—	A	T	T	0.218	0.78 (0.47-1.29)	0.33
10 (rare)	—	—	—	—	—	∗	∗	∗	0.0016	<0.00 (-)	1

Rare: pooled rare haplotypes; OR: odds ratio; CI: confidence interval; p: significance level when *p* < 0.05; SNP1: rs1859430; SNP2: rs2069870; SNP3: rs11741137; SNP4: rs2069885; SNP5: rs2069884; SNP6: rs1800871; SNP7: rs1800872; SNP8: rs1800896.

**Table 8:** Serum IL-10 levels in relation to the genotypes.

Model		Early AMD (pg/ml), median (IQR)	*p* value	Exudative AMD (pg/ml), median (IQR)	*p* value	Atrophic AMD (pg/ml), median (IQR)	*p* value	Control group (pg/ml), median (IQR)	*p* value
*IL-10* rs1800871									
Dominant	GA+AA vs. GG	9.2 (15.8) vs. 9.1 (1.5)	0.571	8.6 (3.9) vs. 7.7 (2.6)	0.312	9.3 (1.1) vs. 9.5 (2.2)	0.230	8.7 (2.6) vs. 8.8 (3.9)	0.442
Recessive	AA vs. GA+GG	(-) vs. 9.2 (1.4)	—	(-) vs. 7.9 (2.9)	—	(-) vs. 9.4 (1.6)	—	(-) vs. 8.8 (2.9)	—

*IL-10* rs1800872									
Dominant	GT+TT vs. GG	9.2 (15.8) vs. 9.1 (1.5)	0.571	8.6 (3.9) vs. 7.7 (2.6)	0.312	9.3 (1.1) vs. 9.5 (2.2)	0.230	8.7 (2.6) vs. 8.8 (3.9)	0.442
Recessive	TT vs. GT+GG	(-) vs. 9.2 (1.4)	—	(-) vs. 7.9 (2.9)	—	(-) vs. 9.4 (1.6)	—	(-) vs. 8.8 (2.9)	—

*IL-10* rs1800896									
Dominant	TC+CC vs. TT	9.1 (1.8) vs. 9.3 (7.8)	0.800	7.2 (2.3) vs. 9.3 (1.4)	**0.048**	9.4 (2.2) vs. 9.3 (0.7)	0.800	8.8 (3.4) vs. 8.7 (-)	0.573
Recessive	CC vs. TC+TT	9.2 (-) vs. 9.1 (2.7)	0.546	6.7 (2.2) vs. 8.5 (2.5)	0.054	8.7 (1.5) vs. 9.5 (1.2)	0.153	8.7 (-) vs. 8.8 (2)	0.958

^∗^Only two genotypes were determined.

## Data Availability

Data will be provided in case a request is made by editors, reviewers, or scientists.

## Supplementary Materials

Supplementary Materials Table S1: genotype distribution in the control group using Hardy-Weinberg equilibrium. Seven SNPs were in HWE (*p* > 0.05), but rs2069870 did not fulfill the HWE requirements because there were observed only two genotypes.
