# Supplementary material for: IL-9 and IL-10 Single-Nucleotide Variants and Serum Levels in Age-Related Macular Degeneration in the Caucasian Population
Source: Mediators Inflamm. 2021 Apr 12;2021:6622934. doi: 10.1155/2021/6622934 (PMC8057879; doi:10.1155/2021/6622934)
Supplement: Supplementary Materials — Table S1: genotype distribution in the control group using Hardy-Weinberg equilibrium. Seven SNPs were in HWE (p > 0.05), but rs2069870 did not fulfill the HWE requirements because there were observed only two genotypes. [file 6622934.f1.docx]

***Supplementary material***

***Table S1. Genotype distribution in the control group using Hardy-Weinberg equilibrium***

| **Gene** | **SNP** | **Allele frequencies** | | **Observed genotype distributions** | **Expected genotype distributions** | **Chi^2^** | **p value** |
| --- | --- | --- | --- | --- | --- | --- | --- |
| *IL-9* | rs1859430 | A (0.21) | G (0.79) | 14/136/233 | 17.6/128.9/236.6 | 1.166 | 0.280 |
| *IL-9* | rs2069870 | G (0.16) | A (0.84) | 0/121/160 | 13/94.9/173 | 21.154 | **<0.001** |
| *IL-9* | rs11741137 | T (0.19) | C (0.81) | 10/126/247 | 13.9/118.2/250.9 | 1.680 | 0.195 |
| *IL-9* | rs2069885 | A (0.19) | G (0.81) | 10/123/250 | 13.3/116.3/253.3 | 1.269 | 0.260 |
| *IL-9* | rs2069884 | T (0.19) | G (0.81) | 10/123/250 | 13.3/116.3/253.3 | 1.269 | 0.260 |
| *IL-10* | rs1800871 | A (0.22) | G (0.78) | 18/133/232 | 18.6/131.7/232.6 | 0.037 | 0.848 |
| *IL-10* | rs1800872 | T (0.22) | G (0.78) | 18/133/232 | 18.6/131.7/232.6 | 0.037 | 0.848 |
| *IL-10* | rs1800896 | C (0.47) | T (0.53) | 77/203/103 | 83.2/190.6/109.2 | 1.616 | 0.204 |

p-significance level, p is statistically significant when <0.05.
